# Morphology and Microstructure Evolution of Gold Nanostructures in the Limited Volume Porous Matrices

**DOI:** 10.3390/s20164397

**Published:** 2020-08-06

**Authors:** Dzmitry V. Yakimchuk, Victoria D. Bundyukova, Jon Ustarroz, Herman Terryn, Kitty Baert, Artem L. Kozlovskiy, Maxim V. Zdorovets, Soslan A. Khubezhov, Alex V. Trukhanov, Sergei V. Trukhanov, Larissa V. Panina, Grigory M. Arzumanyan, Kahramon Z. Mamatkulov, Daria I. Tishkevich, Egor Y. Kaniukov, Vladimir Sivakov

**Affiliations:** 1 Cryogenic Research Division, Scientific-Practical Materials Research Centre, NAS of Belarus, 220072 Minsk, Belarus; yakimchuk@physics.by (D.V.Y.); victoria.bundyukova@gmail.com (V.D.B.); truhanov86@mail.ru (A.V.T.); truhanov@ifttp.bas-net.by (S.V.T.); dashachushkova@gmail.com (D.I.T.); 2 Department Materials and Chemistry, Vrije Universiteit Brussel, Boulevard de la Plaine 5, 1050 Brussels, Belgium; Jon.Ustarroz@vub.ac.be (J.U.); Herman.Terryn@vub.be (H.T.); kitty.baert@vub.be (K.B.); 3 ChemSIN—Chemstry of Surfaces, Interfaces and Nanomaterials, Université libre de Bruxcelles, Campus de la Plaine, Boulevard du Triomphe 2, CP 255. 1050 Brussels, Belgium; 4 Laboratory of Solid State Physics, The Institute of Nuclear Physics, Ibragimov Str. 1, Nur-Sultan 050032, Kazakhstan; artem88sddt@mail.ru (A.L.K.); mzdorovets@gmail.com (M.V.Z.); 5 Laboratory of Engineering Profile, L.N. Gumilyov Eurasian National University, Mirzoyan Str. 2, Nur-Sultan 010008, Kazakhstan; 6 Department of Intelligent Information Technologies, Ural Federal University, Prospekt Lenina 51, 620002 Yekaterinburg, Russia; 7 Department of Physics, North-Ossetian State University, Vatutina Str. 46, 362025 Vladikavkaz, Russia; soslan.khubezhov@gmail.com; 8 Department of Physics and Engineering, ITMO University, Kronverkskiy Prospekt, St. 197101 Petersburg, Russia; 9 Laboratory of Single Crystal Growth, South Ural State University, Lenin prospekt, Chelyabinsk 76, 454080 Chelyabinsk, Russia; ka.egor@mail.ru; 10 Department of Electronic Materials Technology, National University of Science and Technology MISiS, 119049 Moscow, Russia; drlpanina@gmail.com; 11 Department of Raman Spectroscopy (Centre “Nanobiophotonics”), Joint Institute for Nuclear Research, 6 St. Joliot-Curie, 141980 Dubna, Russia; arzuman@jinr.ru (G.M.A.); hero170184@mail.ru (K.Z.M.); 12 Faculty of Nanotechnologies and New Materials, Dubna State University, Ulitsa Universitetskaya, 19, 141982 Dubna, Russia; 13 Departament of Functional Interfaces, Leibniz Institute of Photonic Technology, Albert-Einstein-Str. 9, 07745 Jena, Germany

**Keywords:** gold, nanostructures, template synthesis, X-ray diffraction, X-ray photoelectron spectroscopy, growth mechanism, SERS

## Abstract

The modern development of nanotechnology requires the discovery of simple approaches that ensure the controlled formation of functional nanostructures with a predetermined morphology. One of the simplest approaches is the self-assembly of nanostructures. The widespread implementation of self-assembly is limited by the complexity of controlled processes in a large volume where, due to the temperature, ion concentration, and other thermodynamics factors, local changes in diffusion-limited processes may occur, leading to unexpected nanostructure growth. The easiest ways to control the diffusion-limited processes are spatial limitation and localized growth of nanostructures in a porous matrix. In this paper, we propose to apply the method of controlled self-assembly of gold nanostructures in a limited pore volume of a silicon oxide matrix with submicron pore sizes. A detailed study of achieved gold nanostructures’ morphology, microstructure, and surface composition at different formation stages is carried out to understand the peculiarities of realized nanostructures. Based on the obtained results, a mechanism for the growth of gold nanostructures in a limited volume, which can be used for the controlled formation of nanostructures with a predetermined geometry and composition, has been proposed. The results observed in the present study can be useful for the design of plasmonic-active surfaces for surface-enhanced Raman spectroscopy-based detection of ultra-low concentration of different chemical or biological analytes, where the size of the localized gold nanostructures is comparable with the spot area of the focused laser beam.

## 1. Introduction

In the recent years, the usage of gold, silver, and copper nanostructures for surface-enhanced Raman spectroscopy (SERS) has aroused a lot of interest [1,2,3,4,5,6,7,8] due to the appearance of surface plasmon resonance in these nanostructures when exposed to electromagnetic radiation in the visible and/or near-IR spectral range [1]. The exact position of the plasmon absorption band is determined by the type/nature of the applied metal and the size and shape of the nanostructures [9]. 

The coupled plasmon resonance deserves special attention, as it appears in the gap between nanostructures and leads to the appearance of so-called “hot spots”—areas with a high intensity of local electric fields [9,10,11]. Due to this effect, the highest degree of amplification of the Raman signal can be observed on aggregated structures with different morphology: from dimers [10,12] with one “hot spot” to fractal structures [13,14,15,16,17,18], where a large number of “hot spots” can be achieved. However, the aggregation of nanostructures is often very arbitrary, which leads to broad variation in the “hot spots” average quantity from one aggregate to another. Consequently, the application of aggregated nanostructures for SERS is often avoided. In most cases, the surface active substances are applied to avoid aggregation [19,20] and increase the stability of colloidal plasmonic nanoparticles solutions. The presence of surfactants in colloidal solutions leads to the appearance of additional vibrational modes in the resulting Raman spectrum, which makes it difficult or impossible to correctly interpret the SERS spectra of analyte. Another negative impact of surfactants is related to the creation or increase of an electrostatic barrier in the near-surface area, which leads to a decrease in the adsorption of the analyte molecules and therefore the decrease of signal intensity [21]. 

The using of stabilizers can be avoided by the controlled aggregation of nanostructures on the solid substrates. Mettela et al. have proposed a promising approach to the formation of silver nanostructures with repetitive dendritic-like morphology, which allows the realization of Raman signal with high amplification [22]. However, the synthesis stage involves the utilizing of complex organometallic compounds. It increases the number of operation steps during the proposed synthesis, including high-temperature annealing for the organic matter removal. These are the main disadvantages of such nanostructures’ formation.

A simple method based on the application of a porous matrix for the controlled and localized formation of complex shape plasmonic metal nanostructures has been proposed in our previous publications [23,24,25]. It was shown that the morphology of aggregated structures of copper and silver can be controlled over the large silicon surfaces via galvanic replacement. At the same time, a porous silicon dioxide matrix can be used as a template [26]. Interestingly, the shape of plasmonic metal aggregates is largely influenced by the pore size, which determines the morphology of the deposits, as was found in our previous publication [25]. The subtle adjustment of the unit metal shape can be also carried out by changing the synthesis temperature [23].

Considering the fact that silver and copper nanostructures have low long-term chemical stability against oxidation at ambient atmospheric conditions, especially in water-based solutions, the implementation of gold nanostructures is more realistic. In our previous work, we have already demonstrated the possibility of obtaining gold nanostructures by means of template synthesis [27]. It is important to note that the proposed synthesis method uses "simple" chemistry to produce plasmonic-active nanostructures, which allows us to avoid the consumption of organic additives during the synthesis, and as a consequence, leads to low contamination of the gold surface with derivatives of hydrocarbons. Herein, we present the detailed study of the gold nanostructures’ morphology and microstructure evolution during the selected formation times. To assess the quality of the achieved surfaces, the evolution of the surface states and chemical composition of the resulting gold nanostructures was studied by X-ray photoelectron spectroscopy (XPS). At the same time, SERS’s ability to observe gold-nanostructured surfaces was examined by using a model 2-Mercaptobenzothiazole analyte.

## 2. Materials and Methods

**Figure 1:**
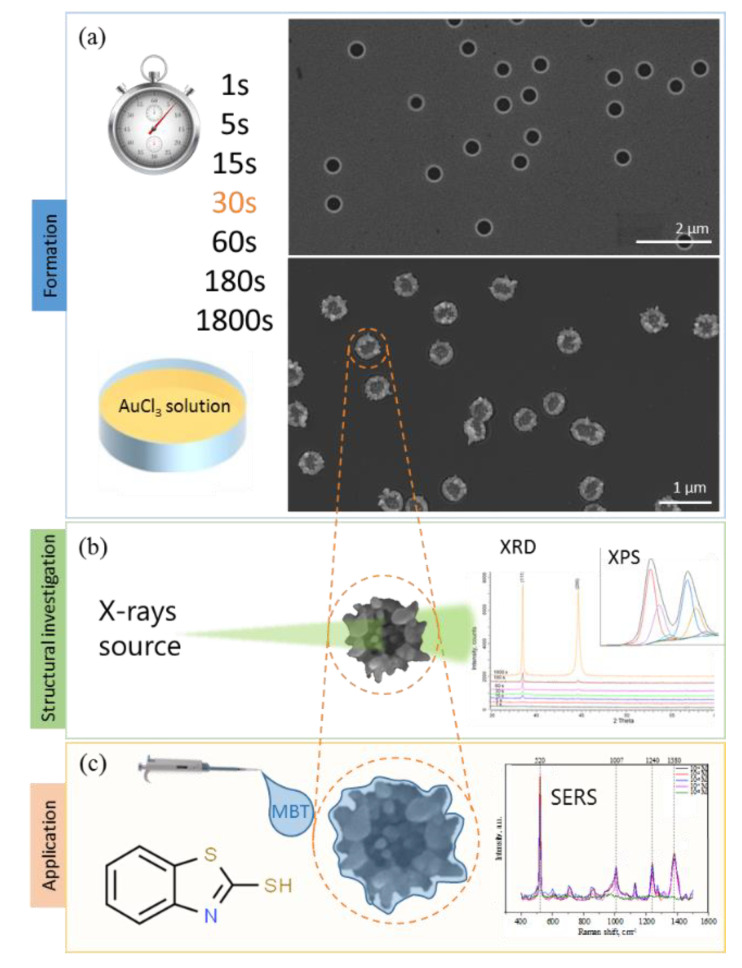
Schematically representation of general idea of formation (**a**), investigation (**b**) and application (**c**) of gold nanostructures in SiO_2_/Si porous matrix.

To obtain the porous SiO_2_/Si matrix, we used the swift heavy ions-track technology [26], which involves the irradiation of the silica surface with high energy ions with the subsequent silica wet-chemical etching of the formed latent tracks in the presence of a selective etching agent. SiO_2_/Si ion-track matrix, in the present study, with the initial pore diameters of 680 nm and 5% surface porosity was used as a template for self-organization of Au clusters in pores with a limited volume. A detailed description of the methods used in the work is given below. The obtained SiO_2_(Au)/Si heterostructures were comprehensively studied for the estimation of the possible growth mechanism of gold nanostructures in porous matrix SiO_2_/Si. The general idea of formation, investigation and application of obtained SiO_2_(Au)/Si heterostructures is shown in Figure 1.

The galvanic replacement method has been used for the formation of gold nanostructures in the pores of the SiO_2_ layer from an aqueous solution of 0.01M gold(III) chloride trihydrate (AuCl_3_·3H_2_O, Sigma-Aldrich) and 5M hydrofluoric acid (HF) during different treatment times from 1 s to 1800 s. The process temperature was stabilized at 298K. The electrolyte before the deposition process has been thermostated in a water bath for 20 min. At the end of the process, the samples were washed in distilled water and dried in a stream of nitrogen.

The chemical transformations during the gold nanostructures growth in SiO_2_/Si pores matrix can be represented as follow, in water, AuCl_3_ and HF dissociate into cations and anions Au^3+^, Cl^−^, H^+^, F^−^, which participate in subsequent chemical reactions, and can be presented as follow:

(1) $$4Au^{3+}+3Si+18F^{-}\to 4Au+3SiF_{6}^{2-},Si+6HF\to H_{2}SiF_{6}+4H^{+}+4e^{-}$$

(2) $$Si+2H_{2}O\to SiO_{2}+4H^{+}+4e^{-},Si+2H_{2}O\overset{HCl}{\to }SiO_{2}+2H_{2}$$

(3) $$SiO_{2}+6HF\to H_{2}SiF_{6}+2H_{2}O$$

The morphology of the observed gold nanostructures was characterized using a field-emission JEOL JSM-7000F scanning electron microscope (SEM).

To investigate the crystallinity of gold precipitates, X-ray diffraction analysis (XRD) was performed on a D8 ADVANCE ECO diffractometer (Bruker, Germany) using Cu K_α_ radiation. To identify the phases and study the crystal structure, the software Bruker AXSDIFFRAC.EVAv.4.2 and the international database ICDD PDF-2 were applied. 

The chemical states of the formed nanostructures were determined by surface-sensitive XPS analysis in an ultrahigh-vacuum (10^−9^ mbar) using K-Alpha ThermoScientific system with a monochromatic X-ray Al Kα 1486.6 eV source. An energy scale was adjusted in accordance with Au 4f = 84 eV, Ag 3d = 368.2 eV, and Cu 2p = 932.6 eV lines of standard samples. To neutralize the surface charge, a calibrated (C 1s = 284.8 eV) compensating electron flood gun was used. The applied X-ray beam spot size was 100 µm. High-resolution spectra were obtained with a spectral resolution of 0.1 eV and constant pass energy of 20 eV. The number of applied scans was 10. For the deconvolution and the component analysis Shirley/Tougard background was used. The line shape of the plotted curves was obtained at a 30% ratio of the Gauss/Lorenz mixture.

The SERS measurements were performed with a LabRAM HR Evolution from Horiba Scientific equipped with a confocal microscope and multichannel air-cooled CCD detector. The laser excitation is provided by the red laser (HeNe: 633 nm; 1−10 mW). A spectral resolution of ~2 cm^−1^ is obtained by using the LWD objective (50×) combined with a grating of 600 gr/mm. To acquire an averaged spectrum from different positions, the laser is rapidly scanned across a defined area on the sample surface (30 s). DuoScanTM (Horiba) is a unique hardware module to scan the laser beam using software-controlled mirrors. The SiO_2_/Si template (4 × 4mm^2^) was placed on a microscope slide with a cavity and 200 µl of 2-Mercaptobenzothiazole (MBT) water solution with different dilutions (10^−4^ M–10^−8^ M) was added and covered by a thinner glass slip. SERS studies were carried out in wet-drop conditions.

## 3. Results and Discussion

### 3.1. Gold Nanostructures’ Morphology

**Figure 2:**
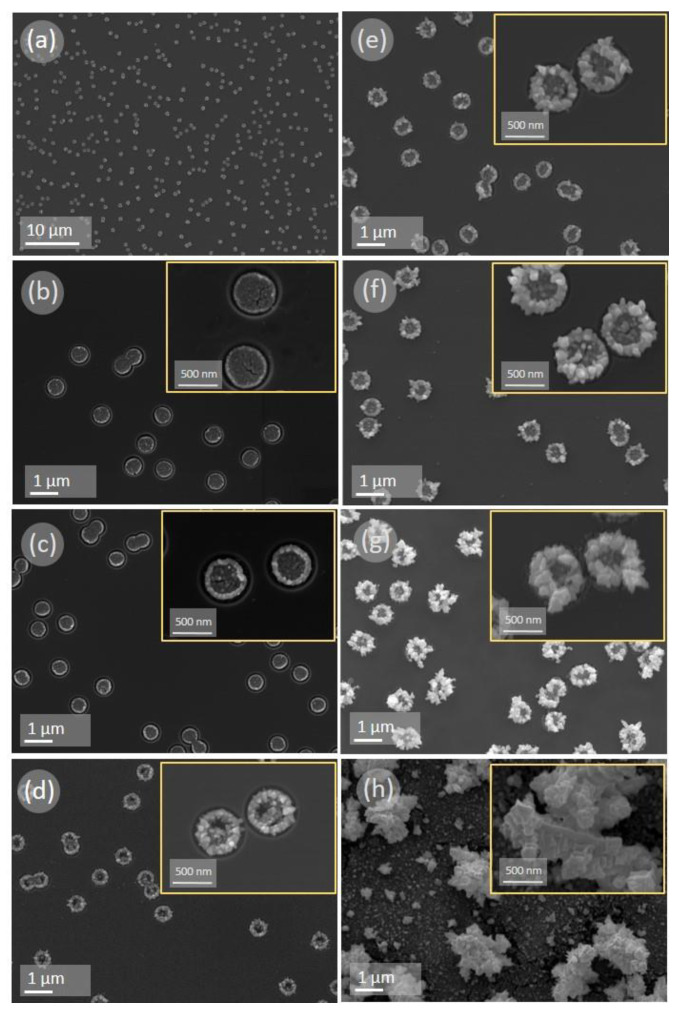
Scanning electron microscope-image planar views of gold nanostructures grown into porous SiO_2_/Si matrix observed at different gold deposition times: (**a**) general planar overview of the surface at deposition time of 30 s; (**b**) 1 s, (**c**) 5 s, (**d**) 15 s, (**e**) 30 s, (**f**) 60 s, (**g**) 180 s, (**h**) 1800 s. (**b−h**) insets: zoomed view of single pores filled with gold agglomerates.

The typical SEM images of gold aggregates obtained via galvanic replacement, at different growth times, in the porous SiO_2_/Si matrix are shown in Figure 2. As it can be seen, at all deposition times, with the exception of the sample at the high deposition time of 1800 s, a high selectivity of the deposition process is presented. A metallic deposit is formed exclusively and localized in the SiO_2_ pores. The gold precipitate is formed in all the pores and all the deposits over the entire substrate have a similar morphology at a given deposition time. The spatial distribution of nanostructures is governed by the location of the pores in the matrix. The time evolution analysis of the morphology of the gold aggregates shows that, even within a shorter time, a dense metal precipitate is formed inside the pore. During the first few seconds, gold particles with diameter lower than 10 nm are formed at the bottom of the pore, as shown in Figure 2b. Characteristically, larger particles are located mostly on the edges of the pores rather in the center of the pores. This may be due to the fact that the initial nucleation occurs more intensely on the defective edge regions in the pores at the SiO_2_/Si interface. After 5 s, an increase in the amount of gold deposits occurs and individual gold crystallites are found at both the edges and in the center of the pores. Typically, particles localized at the edges of the pores increase in size faster than in the central part of the pores, as presented in Figure 2c. The metallic precipitate takes the form of “sunflower-like” structures, similar to our previously published results on the localized silver growth in porous matrices [25], in samples grown at longer than 5 s deposition time, as shown in Figure 2d−g. At longer than 15 s growth time, a gradual increase of the metal deposit can be achieved. The growth of gold nanostructures occurs in a direction perpendicular to the surface, with the formation of aggregated structures mainly at the edges of the SiO_2_ pores. This “sunflower-like” morphology is preserved up to 180 s of deposition time, as shown in Figure 2d−g. However, further increase in the deposition time up to 1800 s leads to both an increase in gold precipitants size and a significant change in their morphology. Observed structures are transformed from selectively and localized “sunflower-like” shapes to gold aggregates with an uncontrollable shape and localization at longer deposition times, as shown in Figure 2h. The matrix surface between big gold agglomerates is covered by smaller gold nanoclusters, as presented in Figure 2h. Possible explanation for such behavior is that at longer deposition times silicon oxide matrix restricting the growth of gold nanostructures is etched completely due to the presence of hydrofluoric acid [28]. As a result, the porous matrix does not exist anymore, and the whole silicon surface can be covered with a film of gold nanoparticles.

### 3.2. X-Ray Diffraction of Gold Nanostructures

**Figure 3:**
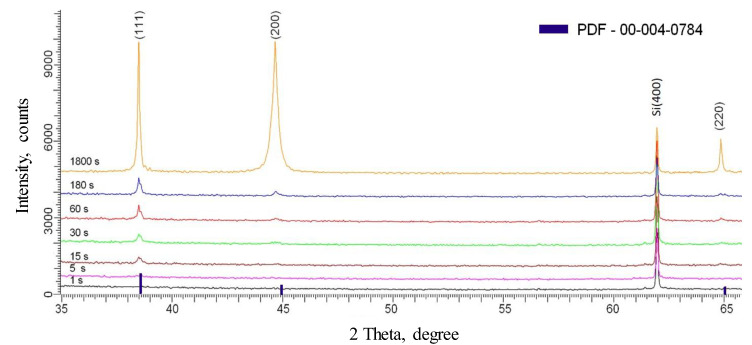
XRD patterns of the porous SiO_2_/Si matrix filled with gold nanostructures at different growth times.

The microstructure of the obtained precipitates was investigated by X-ray diffraction. XRD patterns of gold deposits after different growth times are shown in Figure 3.

At gold deposition times of 1 s and 5 s, the crystalline phase is not presented and amorphous behavior was observed. The absence of X-ray reflexes for the surfaces obtained at a deposition time of 1 s and 5 s is due to the fact that gold atoms do not have enough time to form atomic order in a crystal lattice in such short growth time. This means that deposited gold into the porous matrix is presented either in an amorphous state, or the amount of metal formed during such short deposition times does not reach the sensitivity threshold of the XRD detector. The characteristic gold crystal structure of the face-centered type with the space group Fm-3m (PDF[00-004-0784]) with selected crystallite orientation along the plane (111) was observed for longer than 15 s growth time. 

The calculation of the lattice parameter in the observed samples was carried out using the Nelson–Taylor extrapolation function as presented in Equation (4):

(4) $$a=f\left[\frac{1}{2}\left(\frac{\cos^{2}\theta }{\sin\theta }+\frac{\cos\theta }{\theta }\right)\right]$$

The value and error of the parameter *a* are determined by linear extrapolation of the function to the argument zero value (θ = 90°). 

The average crystallite size can be calculated by the Scherer Equation (5):

(5) $$\tau =\frac{k\lambda }{\beta cos\theta }$$

where *k* = 0.9 is the dimensionless coefficient of the particle shape (Scherer constant), *λ* = 1.54 Å is the X-ray wavelength, β is the half-width, half-height reflex (FWHM) and θ is the diffraction angle (Bragg angle). The calculation results of crystallographic parameters of observed gold nanostructures localized in the porous SiO_2_/Si matrix are given in Table 1.

**Figure 4:**
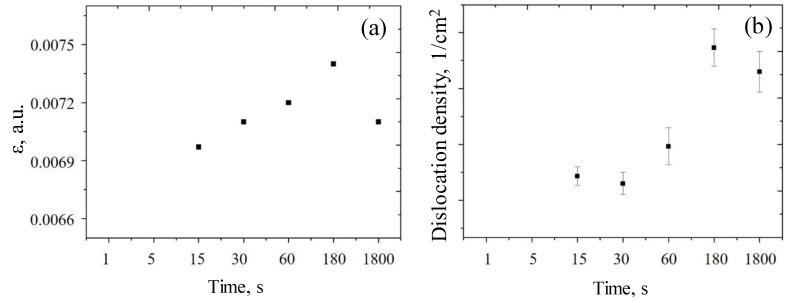
The selected material characteristic dependence on the gold deposition time: (**a**) strain of the crystal lattice in observed nanostructures; (**b**) dislocation density in the deposited nanostructures.

The results presented in Table 1 show that the increase of the deposition time leads to the modest changes in the deposited gold crystal lattice, which is caused by the nucleation processes and the crystallites consolidation along the (111) crystal direction. However, the increase of deposition time leads to a significant difference in the sizes of crystallites formed in different planes. The change in the shape and intensity of the diffraction lines indicates the presence of stresses and deformations in the structure due to the formation of crystallites that is fully logical for polycrystalline materials. The material stresses or strains (ε) were estimated based on a diffraction shift analysis of the most intense peak (111) with respect to the reference values calculated according to Equation (3):

(6) $$\varepsilon =\frac{d_{exp}-d_{pristine}}{d_{pristine}}$$

where *d*_pristine_, *d*_exp_ are interplanar distances of the reference sample and the experimentally obtained data. The dependence of the stresses in deposited gold on deposition time is presented in Figure 4a.

Gold deposition time causes an increase in the stresses and deformations in the crystal structure of the gold precipitant. This effect can take place due to the crystallite formation processes, which leads to a change in the dislocation density of the structures, which is shown in Figure 4b. An increase in the dislocation density is associated with a change of the crystallite size change during the formation process, as well as to subsequent changes in the interplanar distances and crystal structure strains.

### 3.3. XPS Studies of Obtained Gold Nanostructures

**Figure 5:**
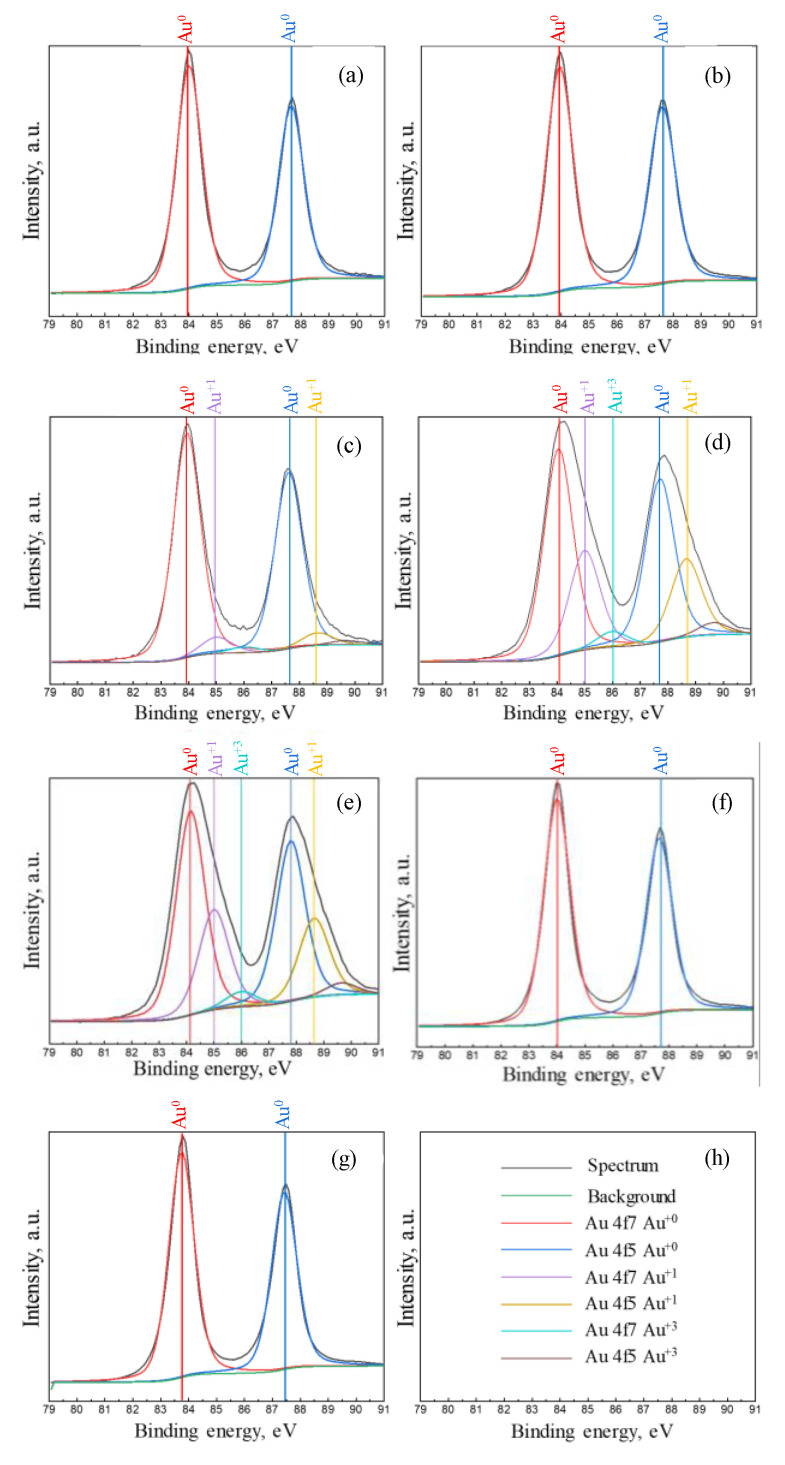
High resolution XPS spectra of Au 4f_7/2_ and 4f_5/2_ core levels in obtained nanostructures grown at different time: (**a**)—1 s, (**b**)—5 s, (**c**)—15 s, (**d**)—30 s, (**e**)—60 s, (**f**)—180 s, (**g**)—1800 s, (**h**)—legend of the graphs.

The results on the obtained surface composition studies by high-resolution XPS are presented in Figure 5. The position and shift of the Au 4f_7/2_ and 4f_5/2_ core levels binding energy values correspond to the presence of different chemical states of gold and can be direct evidence of the presence of different gold oxidation states: Au(0), Au(I), and Au(III) [22,23]. The spectra obtained in Figure 5a,b, are related to the samples with gold deposition times of 1 s and 5 s, where the binding energy values of 87.7 eV and 84 eV are well-correlated to the known binding energy of pure metallic gold(0) [22,23]. The additional binding energy value of the Au 4f at 85 eV (as additional shoulder in spectrum) is related to the presence of the oxidized gold(I) phase on the surface of the gold precipitant grown at 15 s, as shown in Figure 5c. The further increase of deposition time to 30 s and 60 s leads to an increase in the contribution of the Au(I) phase and formation of the additional Au(III) oxidized phase in precipitants, as pointed in Figure 5d,e. For the deposits at higher deposition times of 180 s or 1800 s, only binding energy values characteristic for metallic gold(0) were observed (cf. Figure 5f,g).

**Figure 6:**
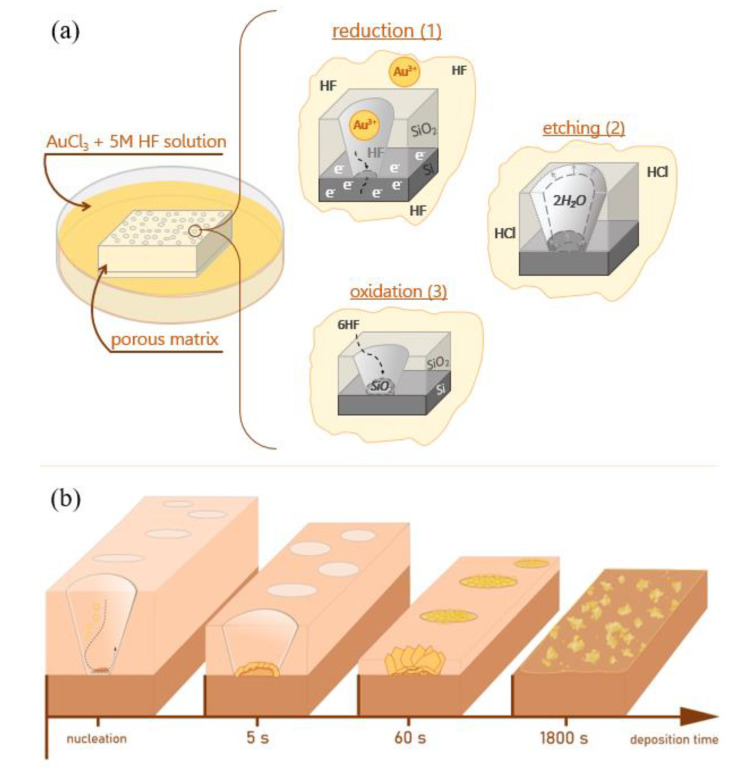
(**a**) Schematic representation of three possible chemical scenarios during the gold precipitant formation in porous SiO_2_/Si matrix; (**b**) evolutions of the morphology of gold nanostructures in the limited volume porous matrices.

XPS data shown in Figure 5, allow us to assume that different growth mechanisms for gold nanostructures may occur at different stages of SiO_2_/Si pore filling. From the analysis of the chemical reaction Equations (1)−(3), it is obvious that three chemical scenarios can take place during the gold deposition: electrochemical reduction of gold on silicon (1), oxidation of silicon (2), and etching of SiO_2_ in fluorine acid (3). The schematic illustration of the pore filling process is demonstrated in Figure 6.

At the initial stage, a metallic gold precipitate is formed by the reduction of gold(III) ions on silicon. In the first stage up to 5 s, the number of electrons pulled out from the silicon surface is in equilibrium with the amount of gold(III) ions, and therefore all ions can be reduced up to a metallic gold(0) state, as was observed in Figure 5a,b. Further, due to the lack of electrons for metal reduction, uncompensated electronic states in gold atoms can be realized. Such behavior can be achieved due to the fact that, in the first initial phase, a dense film of gold nanoparticles had already formed at the bottom of the pore, which blocked hydrofluoric acid access to silicon surface, and as a result not all gold(III) ions can be reduced up to a metallic state. As an evidence of such behavior, the presence of gold ions with lower oxidation level (+1) was observed in the XPS spectrum for the sample grown for 15 s (Figure 5c). In the case of the 30 s and 60 s deposits, the dynamics of incomplete gold ionic reduction continues, which is confirmed by relatively large contributions of ionic states to the resulting gold spectrum (see Figure 5d,e). On this point, we would like to note that the growth of structures continues due to the presence of hydrofluoric acid in the solution, which gradually and evenly erases SiO_2_ pores along the edges, providing access to the silicon surface for the “generation” of new electrons. Thus, there is a diffusion-limited process, and it becomes clear why the structures expand (increase) mainly along the edges of the pores (only in this area do new electrons appear, which instantly can reduce Au(III) ions to Au(0)) [29,30]. However, it can be assumed that the concentration of gold ions in the local region of space at the pore boundaries exceeds the concentration of electrons generated from the silicon surface. A significant part of the generated electrons migrates to the already-formed gold structure. As a result of such behavior, the surface turns out to be negatively charged and gold ions from the solution continue reduction on the surface of the structure, enlarging it. The gold ions, which are around the pore edge and do not receive electrons, form a bond with silicon, as confirmed by XPS spectra performed by Rahsepar and Leung [31]. The formation of Au_3_Si intermetallic alloys at near room temperature have been reported in the 1970s by Bhattacharya et al. [32]. To determine the exact contributions of Au(I) and Au(III) to this bond, as well as to verify the exact theory of the formation of gold silicides in the localized regions of the pore edges, additional studies are required using a synchrotron source in correlation with SEM to accurately position the X-ray beam. These studies are planned in the future. Significant increase in gold growth time (180 s and 1800 s) leads to complete etching of SiO_2_ layers between the pores and the release of the silicon surface, so that gold(III) ions can fully be reduced to the metallic state, as evidenced by XPS spectra without additional contribution of another gold oxidation state have been observed (Figure 5f,g).

Nevertheless, it should be noted that the XPS spectra indicate the absence of the formation of any gold bonds with the components of the initial solution from which the gold nanostructures were obtained. The analysis of the surface states of observed gold nanostructures grown at different deposition times make it possible to demonstrate the possibility of producing gold nanostructures with a positively charged surface due to the presence of silicide (Au-Si) in the structure. As mentioned in [33], metal silicides, including gold, could be used as a catalyst. The advantage of these structures over existing ones (as typically colloidal solutions) is that they are localized on a solid substrate.

### 3.4. SERS on Obtained Gold Nanostructures

**Figure 7:**
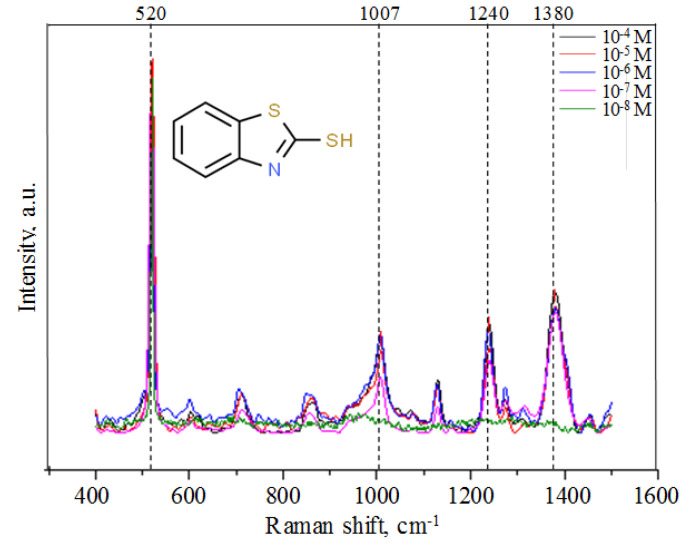
SERS spectra of MBT analyte (inset) with concentrations between 10^−4^ M and 10^−8^ M.

The potential of the realized gold nanostructures as a SERS-active substrate is tested on model analyte, MBT solutions, with different concentrations (10^−4^ M–10^−8^ M). The gold precipitates obtained after a deposition time of 180 s were selected, because they approach the necessary size of the laser spot of about 1 µm. Depending on the measured spot, the intensity of the Raman signal varies, so we used a scanning laser beam, where the area of 25 × 25 µm^2^ is continuously scanned during the Raman scattering measurement. We found that for concentrations of 10^−4^ M to 10^−7^ M the similar Raman spectrum is obtained, as presented in Figure 7.

The high intensity peak in the SERS spectra located at 520 cm^−1^ corresponds to the crystalline silicon in porous matrix, at 1380 cm^−1^ originates from combined stretching of the NCS-ring and peak at 1007 cm^−1^ and 1240 cm^−1^ relates to the C-S stretching modes [34]. It is well-known and previously published by our group on similar nanostructures [27,35], that chemically reactive (e.g., thiol in 2-Mercaptopyridin) groups containing molecules have strong chemical binding with metal surfaces, that can significantly affect SERS signal intensity due to the Raman signal enhancement as a consequence of chemical factor. In present study, SERS spectrum for the lowest MBT concentration of 10^−8^ M cannot be detected that can be explained by the lower MBT molecules cross-linking of the gold nanostructures surface. The direct evidence of such behavior is the decrease of the 1007 cm^−1^ and 1240 cm^−1^ C-S stretching modes intensity, because the MBT molecule binding to the surface take place through –SH group [36] that means that amount of adsorbed or bonded MBT molecules to the gold surface directly correlates with intensity of C-S vibrational modes. Considering the size of the sample and the amount of solution, the solution should contain at least 10^−7^ M of MBT to obtain a monolayer of analyte on the gold agglomerates.

## 4. Conclusions

The localized gold nanostructures were obtained by galvanic replacement in the pores of the SiO_2_/Si matrix. The evolution of microstructural and morphological features of the gold aggregates depending on the time of metal deposition was studied. The deep investigation of the atomic structure and chemical composition of the surface of the samples obtained during deposition from 15 to 60 s allowed us to reveal the contribution of Au(I) and Au(III) gold states in XPS spectra. It is assumed that this fact is caused by an incomplete reduction of gold ions to a metallic state, due to diffusion-limited processes occurring in the pores of the SiO_2_/Si matrix. It should be noted that these SERS-active substrates were tested by being in a usual atmosphere for 1 year, while maintaining their SERS-activity. Thus, the proposed structures are stable at ambient atmospheric conditions for at least a year. Such functional surfaces are well-suited for the SERS-based detection of molecules. Additionally, observed surfaces with different oxidized states of gold have perspectives as promising catalysts.

## Figures and Tables

**Table 1:** The main crystallographic parameters of the crystal structure of the gold precipitants at different crystal growth times.

Deposition Time	Phase	Space Group	(hkl)	Crystallite Size, nm	Cell Parameter, Å	FWHM
1 s	amorph	—	—	—	—	—
5 s	amorph	—	—	—	—	—
15 s	cubic	Fm-3 m(225)	111	88.68	4.05061	0.105
30 s	cubic	Fm-3 m(225)	111	88.00	4.05223	0.106
60 s	cubic	Fm-3 m(225)	111	111.42	4.04901	0.084
			200	53.35		0.179
			220	80.35		0.130
180 s	cubic	Fm-3m(225)	111	88.90	4.04740	0.105
			200	56.74		0.168
			220	65.07		0.161
1800 s	cubic	Fm-3m(225)	111	105.10	4.04901	0.089
			200	34.10		0.280
			220	95.26		0.110
